# UCST-Type Soluble Immobilized Cellulase: A New Strategy for the Efficient Degradation and Improved Recycling Performance of Wastepaper Cellulose

**DOI:** 10.3390/molecules29051039

**Published:** 2024-02-28

**Authors:** Zhaohui Chen, Jiacong Wu, Juan Han, Yun Wang, Liang Ni

**Affiliations:** 1 School of Chemistry and Chemical Engineering, Jiangsu University, Zhenjiang 212013, China; 15751002807@163.com (J.W.); yunwang@ujs.edu.cn (Y.W.); niliang@ujs.edu.cn (L.N.); 2 School of Food and Biological Engineering, Jiangsu University, Zhenjiang 212013, China; hanjuan@ujs.edu.cn

**Keywords:** UCST, immobilized enzyme, cellulase, catalyze, waste-paper cellulose

## Abstract

This paper reports an innovative study that aims to address key issues in the efficient recycling of wastepaper cellulose. The research team utilized the temperature-responsive upper critical solution temperature (UCST) polymer P(NAGA-b-DMA) in combination with the LytA label’s affinity for choline analogs. This innovative approach enabled them to successfully develop a novel soluble immobilized enzyme, P(NAGA-b-DMA)-cellulase. This new enzyme has proven highly effective, significantly enhancing the degradation of wastepaper cellulose while demonstrating exceptional stability. Compared with the traditional insoluble immobilized cellulase, the enzyme showed a significant improvement in the pH, temperature stability, recycling ability, and storage stability. A kinetic parameter calculation showed that the enzymatic effectiveness of the soluble immobilized enzyme was much better than that of the traditional insoluble immobilized cellulase. After the immobilization reaction, the Michaelis constant of the immobilized enzyme was only increased by 11.5%. In the actual wastepaper degradation experiment, the immobilized enzyme was effectively used, and it was found that the degradation efficiency of wastepaper cellulose reached 80% of that observed in laboratory conditions. This novel, thermosensitive soluble immobilized cellulase can efficiently catalyze the conversion of wastepaper cellulose into glucose under suitable conditions, so as to further ferment into environmentally friendly biofuel ethanol, which provides a solution to solve the shortage of raw materials and environmental protection problems in the paper products industry.

## 1. Introduction

In recent years, the paper manufacturing industry has been grappling with the growing scarcity of raw materials alongside a heightened concern for environmental protection. Consequently, there has been a substantial surge in research interest directed towards the effective recycling of cellulose extracted from wastepaper [1]. The efficient repurposing of cellulose from discarded paper products addresses a critical challenge faced by the paper industry concerning its raw material supply. Moreover, it plays an essential role in fostering eco-friendly energy solutions and curbing environmental pollution, thereby contributing significantly to global sustainability efforts [2,3].

As an emerging technology, bioenzymatic catalysts have gained widespread application in the wastepaper processing industry due to their fast reaction rates, mild reaction conditions, and minimal environmental pollution [4,5,6]. Enzymes, commonly employed as biocatalysts, can significantly improve the hydrolysis rate of cellulose and reduce the energy consumption for pollution treatment when utilized in wastepaper pulp processing. However, the high water solubility of free enzymes poses a challenge to their recovery during usage. Moreover, their catalytic activity is confined to a specific range of temperature and pH levels, which restricts their applicability in wastepaper cellulose processing.

Enzymatic immobilization technology encompasses various methods, including covalent binding, adsorption, encapsulation, and cross-linking, to effectively immobilize enzymes onto carrier materials for the facile recovery of enzymes [7,8,9]. Additionally, immobilized enzymes often provide a certain level of protection to maintain high enzymatic activity across a fairly broad range of temperatures and pH values [10]. However, unlike soluble free enzymes, most traditional immobilized enzymes pose challenges in terms of solubility. Challenges such as substrate diffusion and mass transfer resistance frequently arise in enzyme-catalyzed reactions involving solid substrates.

Huang W et al. employed a covalent binding method to immobilize cellulase onto magnetic Fe_2_O_3_/Fe_3_O_4_ nanocomposites. While the nano-carrier materials offer an increased surface area and porosity, partially offsetting the disadvantage of elevated mass transfer resistance, the immobilized enzyme shows a fivefold surge in the Michaelis constant [11]. This suggests a significant reduction in the affinity between the enzyme in its immobilized state and the substrate. Temperature-responsive polymers, when subjected to specific thermal conditions, become soluble. Using them to create immobilized cellulase enables enhanced interactions with substrates, resulting in faster reaction rates. Temperature-responsive polymers are categorized as the lower critical solution temperature (LCST) type or the upper critical solution temperature (UCST) type [12]. LCST-type polymers have been extensively investigated due to their broad range of applications and facile synthesis. However, immobilized enzymes using LCST-type polymers necessitate precipitation and recovery at temperatures higher than the enzyme catalytic temperature, demanding a high thermal stability of the enzyme. Consequently, LCST-type temperature-responsive immobilized enzymes often exhibit a suboptimal cycling utilization performance. In contrast, UCST-type immobilized enzymes can be precipitated and recovered under conditions lower than the catalytic temperature of the enzyme, thereby maximizing the preservation of catalytic activity.

With the advancement of immobilization technology, there has been a noticeable increase in the application of affinity labels for both expressing and immobilizing recombinant enzymes [13,14,15,16]. Affinity labels are proteins possessing distinctive structures that are typically attached to either the N or C terminal of recombinant proteins, thereby facilitating their separation, purification, and immobilization. There exist two primary categories of affinity labels: epitope labels and protein/structural domain labels. Epitope labels refer to small peptides exhibiting a strong affinity towards chromatographic resins, including histidine (His) labels [17,18], FLAG labels [19], hemagglutinin (HA) labels [20], etc. Protein/structural domain labels typically have a higher molecular weight compared to epitope labels and can exert various effects, including promoting gene expression, enhancing the solubility of recombinant proteins, and exhibiting an affinity-enhancing effect known as ligand binding affinity labels. Commonly used protein/structural domain labels include glutathione S-transferase (GST) [21], calmodulin binding peptide (CBP) [22,23], maltose binding protein (MBP) [24,25], and elastin-like peptide (ELP) [26,27]. The choline-binding protein LytA belongs to the category of protein/structural domain labels and was initially discovered in Streptococcus pneumoniae. It exhibits high affinity binding to choline residues attached to the cell wall surface [28,29]. This choline-binding protein demonstrates a strong affinity for choline and choline structural analogues [30] and can serve as an effective affinity label for enzymatic immobilization [31,32].

In this experiment, a soluble immobilized enzyme preparation method was applied to the immobilization of cellulase, an enzyme with a solid substrate, using the affinity of LytA labels and choline-like substances to prepare a soluble UCST temperature-responsive bioenzymatic catalyst P(NAGA-b-DMA)-cellulase. By leveraging the affinity between the LytA labels and choline analogs, the immobilized enzyme dissolves at elevated temperatures, rapidly catalyzes reactions with the substrate, and can be efficiently reclaimed through cooling. This approach ensures the preservation of the maximum enzymatic activity and significantly enhances its recyclability. The liquid–solid phase catalysis rate between the soluble immobilized cellulase and the substrate cellulose is significantly better than that between the traditional insoluble immobilized enzyme and insoluble substrate. An analysis using kinetic metrics shows a significant improvement in the substrate interaction for the enzyme immobilized through this method over conventional insoluble immobilized cellulases. This immobilized enzyme exhibits a superior temperature stability, pH stability, storage stability, and cycling use stability compared to free enzymes. Ultimately, the successful application of the immobilized enzyme was achieved in the hydrolysis experiments involving actual wastepaper cellulose.

## 2. Results and Discussion

**Figure 1:**
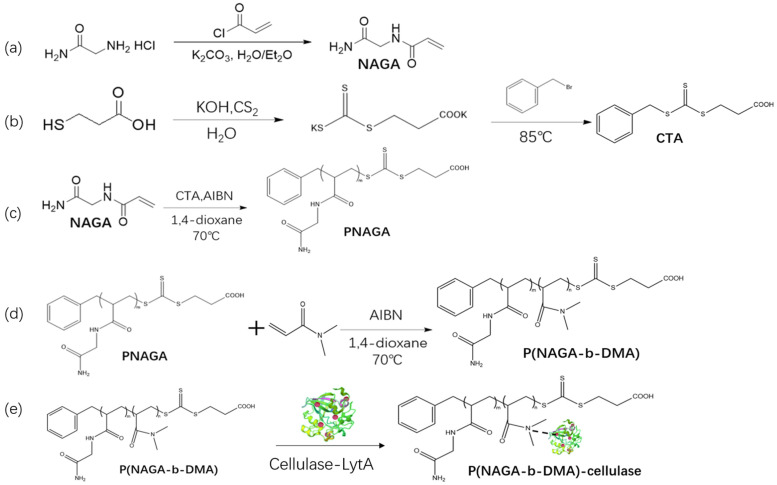
Synthesis process of P(NAGA-b-DMA)-cellulase. (**a**) Synthesis process of NAGA. (**b**) Synthesis process of CTA. (**c**) Synthesis process of PNAGA. (**d**) Synthesis process of P(NAGA-b-DMA). (**e**) Synthesis process of P(NAGA-b-DMA)-cellulase.

Synthesis and characterization of P(NAGA-b-DMA): Figure 1 demonstrates the synthesis process in our study, where we created a UCST-type temperature-responsive block copolymer, P(NAGA-b-DMA) by utilizing reversible addition–fragmentation chain-transfer (RAFT) polymerization on NAGA and DMA monomers. The NAGA segments impart the UCST characteristic to the copolymer. The choline-binding protein LytA, a 135-amino-acid repeat sequence protein, possesses numerous choline binding domains with a high affinity for choline moieties, demonstrating a strong attraction towards both choline and its structural analogs [28,29]. In this experiment, the affinity between cellulase with an LytA label and DMA with a choline-like structure was used to achieve the immobilization of cellulase. As depicted in Figure S1, the reaction products of each step during the synthesis were checked using ^1^H NMR to ensure the completion of the reaction.

Characterization: In this experiment, six UCST polymers were synthesized with varying degrees of NAGA and DMA polymerization to examine the influence mechanism of different monomer polymerization degrees on polymer UCST. The molecular weights and dispersion coefficients of these polymers are presented in Table 1. The molecular weight of the polymer as assessed using gel permeation chromatography (GPC) showed a negligible deviation from the theoretical molecular weight, essentially aligning with the anticipated trend. The results indicate that the synthesized polymers exhibit a high reliability.

**Figure 2:**
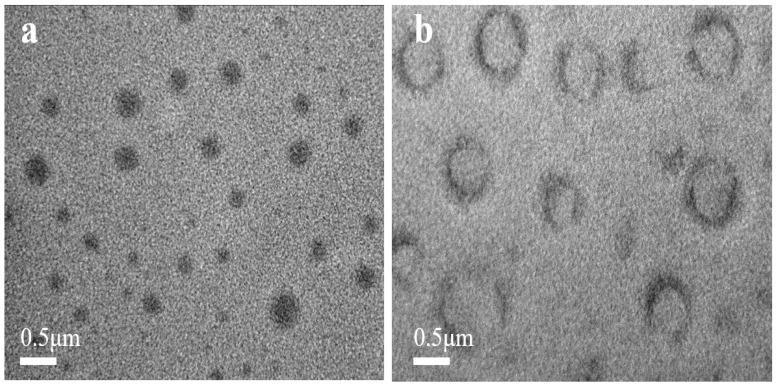
TEM data of P(NAGA-b-DMA) (**a**) above the UCST and (**b**) below the UCST.

Observations were made on polymer solutions above or below the UCST temperature using transmission electron microscopy (TEM), as depicted in Figure 2. At temperatures higher than the UCST, the polymer exhibits spherical black nanoparticles with diameters of 200–400 nm. When the temperature falls below the UCST, the polymer nanoparticles have a diameter of approximately 1000 nm and display a black outer ring with a white interior. This is because of the disruption of hydrogen bonds within the polymer molecules at high temperatures, causing the expansion of the polymer chains and allowing for the phosphotungstic acid solution to penetrate into the entire polymer structure, resulting in its black appearance. When the temperature is below the UCST, the polymer forms intramolecular hydrogen bonds, causing the polymer chains to contract tightly, greatly increasing the hydrophobicity. Water-soluble phosphotungstic acid cannot penetrate into the interior of the polymer molecule and can only cover its surface. Therefore, at this time, a black outline can be observed on the surface of the polymer, but the interior is not stained or blackened. At this juncture, the augmentation in particle size can be attributed to the development of a polymer framework structure influenced by hydrogen bonds, similar to the phenomenon that ice is larger than liquid water [33,34].

The particle size distribution in polymer solutions below or above the UCST was observed through dynamic light scattering, as depicted in Figure S2. The particle size of the solution below the UCST temperature measures approximately 300 nm, whereas the particle size of the polymer solution above the UCST temperature is approximately 1000 nm. These experimental outcomes are consistent with the TEM observations, thus collectively verifying the manifestation of UCST characteristics in the polymer.

Study on the UCST properties of P(NAGA-b-DMA): In this experiment, the effects of the polymer solution concentration, NAGA block polymerization degree, DMA block polymerization degree, and urea concentration in solution on the UCST properties of the carrier polymer were studied, respectively, to prepare for the next enzyme immobilization experiment.

As depicted in Figure S3a, the UCST of the solution increases notably as the polymer solution concentration rises. This occurs because when the concentration of the polymer solution is augmented, the density of the polymer molecules within a unit volume of the solution significantly increases. As a result, each polymer molecule has a higher likelihood of interacting with others, leading to the formation of more hydrogen bonds among the polymer chains. These additional hydrogen bonds require extra energy to break them before allowing the polymer to continue dissolving in water, hence resulting in an observed increase in the UCST [35].

As depicted in Figure S3b, in the comparison of the dissolution curves for polymer UCST1-3, the UCST of the polymer solution continuously increases with a rise in the NAGA polymerization degree. This is due to the presence of more amide groups in the polymer with a higher degree of NAGA polymerization degrees, allowing the formation of more intramolecular and intermolecular hydrogen bonds. The augmented intramolecular hydrogen bonding results in the tighter packing and contraction of individual polymer chains, which impedes water penetration and enhances the overall hydrophobicity. Meanwhile, the increased intermolecular hydrogen bonding between polymer molecules leads to the assembly of more stable spherical aggregates. The disassociation of these tightly bonded spheres necessitates higher temperatures, thereby resulting in an elevated UCST for the polymer solutions [36].

As depicted in Figure S3c, with the increase in the DMA block content of the polymer, the UCST of the polymer solution continues to decrease, although not significantly. Despite having a strong hydrophilicity, DMA blocks still constitute a small proportion within the polymer, limiting the extent of the UCST reduction. Additionally, as the degree of DMA polymerization increases, the overall molecular weight of the polymer increases, and there is also a certain degree of increase in the hydrophobicity, which offsets some of the increased hydrophilicity brought by DMA blocks.

Urea solutions of varying concentrations were introduced to the polymer solution to study the influence of altered hydrogen bond compositions on the UCST of the polymer, as illustrated in Figure S3d. The addition of urea continuously lowered the UCST of the solution. As the concentration of urea increased, the polymer solution no longer exhibited a UCST performance, and became completely soluble within a temperature range of 0–100 °C. Due to the presence of amino and carbonyl groups capable of forming hydrogen bonds, when urea is added to the polymer solution, it forms numerous hydrogen bonds with NAGA block on the polymer, resulting in a reduction in both the intramolecular and intermolecular hydrogen bonds within the NAGA block. The reduction in intramolecular hydrogen bonds allows for the polymer chain to stretch out, significantly enhancing the hydrophilicity. The reduction in intermolecular hydrogen bonds prevents the polymer molecules from clustering, and instead disperses them evenly in water. When the urea concentration is sufficiently high, it covers almost all the amino and carbonyl groups on the NAGA block, resulting in too few hydrogen bonds for the polymer to form. The energy in the solution at 0 °C is enough to dissociate these few remaining hydrogen bonds, causing the polymer solution to lose its UCST properties. This experiment demonstrates that the hydrogen bond is the main driving force behind a polymer exhibiting UCST properties [37].

Cultivation and characterization of LytA-labeled cellulase: The molecular weights of the three target enzymes (CBH-linker-LytA, EG-Linker-LytA, and Glu-Linker-LytA) prepared in this experiment are approximately 91, 84, and 89 kDa, respectively. Figure S4 depicts the SDS-PAGE analysis of the mixed enzyme solution obtained by combining equal amounts of crude enzyme solution for all three enzymes in a ratio of 1:1:1. The target enzyme bands within the red box are clearly visible at their expected molecular weights, providing evidence for the presence of the target enzyme in the mixed enzyme solution [38].

Optimization of immobilization conditions for P(NAGA-b-DMA)-cellulase: In this experiment, LytA-labeled cellulase was prepared. The immobilized enzyme P(NAGA-b-DMA)-cellulase was prepared by utilizing the affinity between the LytA label and choline analogs through DMA as an affinity monomer. A series of experiments were conducted to identify the optimal conditions for the immobilization reaction between cellulase and P(NAGA-b-DMA). These experiments aimed to assess the immobilization effectiveness based on two key parameters: the enzyme’s capacity for immobilization and the retention of enzymatic activity in the immobilized form. In the end, the conditions for the immobilization reaction were established as follows: 2 g/L UCST-5 was introduced into 2 mL of the mixed enzyme solution, and the reaction took place at pH 6 and 60 °C for a duration of 70 min. Under these conditions, the maximum immobilization capacity obtained for the enzyme was 212 mg/g. See supporting information for details.

**Figure 3:**
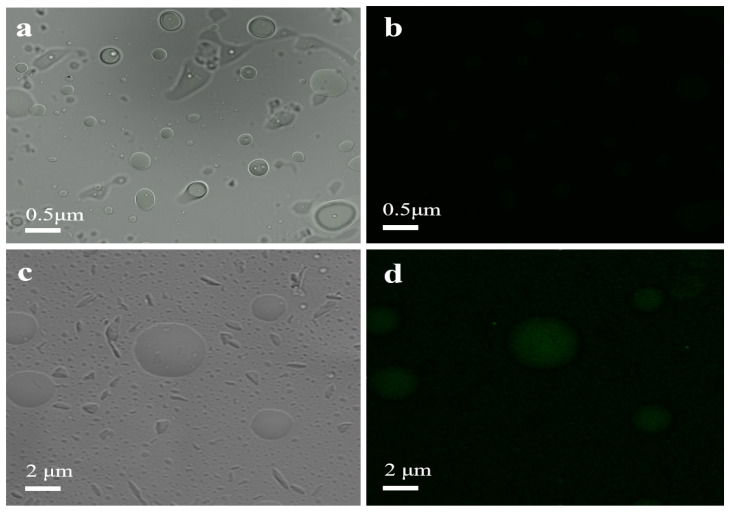
Laser confocal data of P(NAGA-b-DMA) (**a**,**b**) and P(NAGA-b-DMA)-cellulase (**c**,**d**).

Characterization of P(NAGA-b-DMA)-cellulase. Cellulase was labeled with fluorescein isothiocyanate (FITC), and the fluorescence of the polymer and immobilized enzyme before and after the immobilization reaction was observed at an excitation wavelength of 288 nm through a laser confocal microscope to evaluate the effectiveness of the immobilization. The test results of the laser confocal microscope are depicted in Figure 3. In the bright field, numerous spherical particles can be observed in P(NAGA-b-DMA) (Figure 3a), while these particles exhibit minimal fluorescence in the dark field (Figure 3b). In the bright field, the diameter of the spherical particle in P(NAGA-b-DMA)-cellulase increases nearly fivefold (Figure 3c), and the entire sphere displays a consistent green fluorescence (Figure 3d). This demonstrates that cellulase can be stably immobilized on P(NAGA-b-DMA). However, the average molecular weight of LytA-labeled cellulase is approximately 90,000.00 g/mol, which is much higher than the molecular weight of the polymer (15,000–25,000 g/mol). Nonetheless, considering the enzyme’s immobilization capacity, it can be deduced that the polymer content in the immobilized enzyme is notably greater than that of the enzyme alone. Therefore, the primary reason for the increase in the particle sizes of the immobilized enzymes is not due to enzymes themselves, but rather because each enzyme simultaneously undergoes affinity reactions with multiple polymer molecules, resulting in multiple polymer molecules being linked together by a small amount of cellulase molecules, forming a large molecular cluster of immobilized enzymes. Furthermore, the inclusion of cellulase in the immobilization procedure notably elevates the molecular weight of the entire molecular assembly. This contributes to enhancing the UCST of the polymer [39,40].

**Figure 4:**
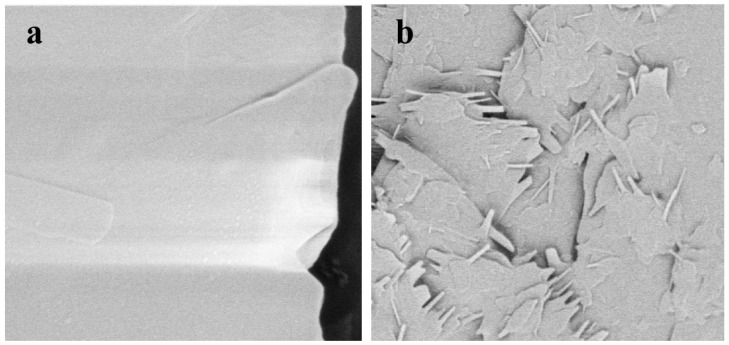
SEM data of P(NAGA-b-DMA) (**a**) and P(NAGA-b-DMA)-cellulase (**b**).

Figure 4 demonstrates the scanning electron microscopy (SEM) images of freeze-dried powder of P(NAGA-b-DMA) (Figure 4a) and P(NAGA-b-DMA)-cellulase (Figure 4b) solutions. After freeze-drying, the polymer’s surface exhibits a relatively smooth, extensive, plate-like layered structure, which can be ascribed to the tightly packed polymer molecules facilitated by hydrogen bonding at lower temperatures, a characteristic that remains well preserved throughout the freeze-drying process. The SEM images of the immobilized enzyme demonstrate that the original layered structure has fragmented into smaller flakes with uneven surfaces and needle-like structures along the edges. The introduction of cellulase does not significantly affect the hydrogen bonds within the polymer molecules, allowing the polymers to maintain a relatively regular arrangement. However, intermolecular hydrogen bonds are greatly affected, preventing the polymers from aggregating into large plate-like areas. The irregular protrusions on the surfaces of the immobilized enzymes also demonstrate the successful immobilization of cellulase [41].

Figure S7 illustrates the results of the thermogravimetric analysis for both P(NAGA-b-DMA) and P(NAGA-b-DMA)-cellulase. In the temperature range of 0–200 °C, both samples show an approximately 15% weight loss, which is attributed to the evaporation of the moisture absorbed in the sample. In the range of 200 to 600 °C, the weight loss of the carrier polymer is typically less than that of the immobilized enzymes. This difference in weight loss between 200 to 600 °C can be attributed to additional degradation due to proteolysis occurring within the immobilized cellulase, thereby providing indirect evidence of its successful immobilization onto the P(NAGA-b-DMA) polymer [42].

Study on the UCST properties of P(NAGA-b-DMA)-cellulase: As shown in Figure S8, the UCST behavior of P(NAGA-b-DMA)-cellulase was basically the same as that of the carrier polymer, and its change trend was the same under all different conditions. The introduction of the enzyme only increased the UCST of the immobilized enzyme. This is because the molecular weight of the enzyme is much larger than that of the carrier polymer, and the introduction of the enzyme molecule significantly increases the molecular weight of the entire immobilized enzyme molecule, resulting in an increase in its hydrophobicity. In this case, more hydrogen bonds need to be broken in order for the immobilized enzyme to dissolve in water, and breaking more hydrogen bonds means that more energy needs to be consumed, so an increase in the UCST is observed [43].

Optimal catalytic conditions of free cellulase and P(NAGA-b-DMA) cellulase: The optimum catalytic conditions for the immobilized enzyme are a temperature 60 °C and pH = 6. See supporting information for details.

Kinetic parameters of free cellulase and P(NAGA-b-DMA) cellulase: In this experiment, the kinetic parameters for the catalysis of free cellulase and P(NAGA-b-DMA)-cellulase on the catalyzing substrate CMC are depicted in Table 2. Figure S10 demonstrates the double reciprocal equation fitting graph for free and immobilized enzymes [44].

According to Table 2, while there is a noticeable reduction in the affinity and catalytic efficiency of the immobilized cellulase towards its substrate, this decrement is not substantial and maintains an approximately similar order of magnitude. This is due to the fact that during the immobilization process, the enzyme’s catalytic active sites are partially covered by the polymer of the carrier. The slight increase in the calculated K_m_ values indicates that the immobilized enzyme prepared using the affinity reaction using a LytA label with a structure similar to choline analog does not significantly disrupt the catalytic active site of cellulase. Table 2 also presents a comparison of the immobilization method for the soluble cellulase enzyme with those used. Mo. et al. used chitosan/magnetic porous biochar as a carrier and covalently immobilized the cellulase using glutaraldehyde [45]. Yan. et al., on the other hand, utilized the physical adsorption of Fe_3_O_4_@C magnetic carbon spheres coupled with S_i_O_2_ encapsulation to physically absorb and immobilize the cellulase [46]. The traditional insoluble immobilization reaction often leads to an increase of more than 50% in the Michaelis constant, but the soluble immobilized enzyme prepared in this experiment only increases by 11.5%. This is due to the fact that the immobilized enzyme used in this study remains liquid at catalytic temperatures, allowing for a liquid–solid phase catalysis with the substrate. Consequently, a marked enhancement in the interaction between the immobilized enzyme and its substrate is observed.

**Figure 5:**
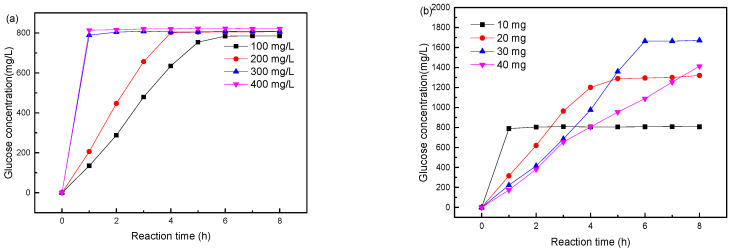
Effects of enzyme concentration (**a**) and substrate addition (**b**) on catalytic performance.

Productivity analysis of immobilized cellulases: In this experiment, the yield of the whole reaction process under the optimal catalytic condition was measured by the productivity curve to evaluate the catalytic effect of the immobilized enzyme on the free enzyme [47]. A total of 10 mg of finely crushed qualitative filter paper was introduced into 10 mL of immobilized enzymatic solution, with concentrations ranging from 100 to 400 mg/L. The catalytic reaction was carried out at 50 °C and pH = 6 for 8 h, with the glucose concentration in the solution measured every hour to evaluate the catalytic effect. The experimental findings are illustrated in Figure 5a, revealing that augmenting the quantity of the immobilized enzyme has minimal impact on the reaction limit but significantly enhances the rate of the catalytic reaction. When the mass ratio between the immobilized enzyme to substrate is greater than or equal to 3:10, the reaction can be completed within one hour.

Crushed qualitative filter paper ranging from 10 to 40 mg was added to 10 mL of an immobilized enzyme solution, maintaining a constant concentration of 300 mg/L. The catalytic process was conducted at 50 °C and pH 6 for a duration of 8 h, during which the glucose concentrations were monitored hourly to evaluate the catalytic efficiency. The experimental findings are presented in Figure 5b, indicating that increasing the substrate excessively does not proportionally increase the reaction rate. When the enzyme-to-substrate ratio is 3:10, the reaction limit can be reached within one hour, with 80% of the crushed qualitative filter paper hydrolyzed into glucose. However, as the substrate increases and when the enzyme-to-substrate ratio is 3:20, it takes five hours to reach the reaction limit, resulting in only 65% of the filter paper hydrolyzed into glucose. When the ratio of immobilized enzyme to substrate is 3:40, even after 8 h of catalysis, the reaction has not reached its maximum potential, with only 35% of the filter paper being hydrolyzed into glucose. The decline in cellulose hydrolysis efficiency in high substrate concentration conditions can be attributed to the inhibitory influence of elevated product concentrations on the advancement of the forward reaction [38].

In conclusion, considering the limitations of the reaction rate and reaction time, the optimal catalytic reaction conditions are at 50 °C and pH = 6. The reaction is carried out with 300 mg/L of immobilized enzyme and cellulose substrate in a mass ratio of 3:10. Under these conditions, the reaction can be completed within one hour, with 80% of the substrate being hydrolyzed into glucose. After the reaction, the solution was filtered while still hot, and then left to settle at a low temperature of 4 °C for 20 min before separation and recovery. The enzyme exhibits a recovery rate of 91.7%.

**Figure 6:**
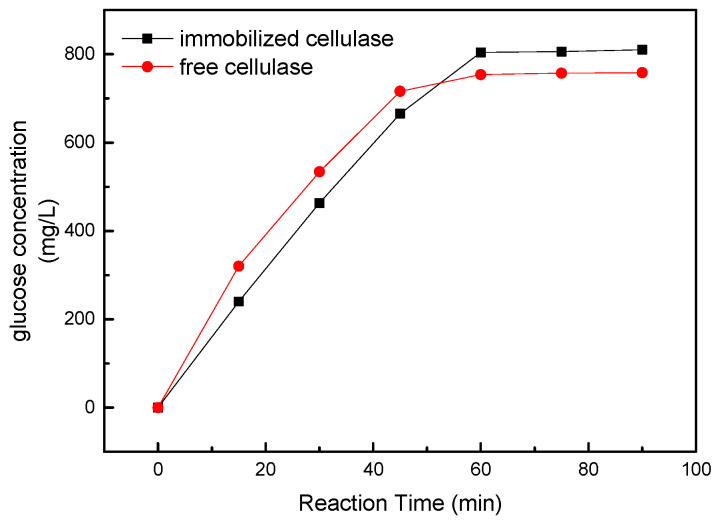
Comparison of catalytic performance between free cellulase and immobilized cellulase.

Under the above optimal catalytic conditions, the catalytic effects of the free enzyme and immobilized enzyme were compared, and the results are shown in Figure 6. While the free cellulase demonstrated a somewhat higher initial catalytic rate, the difference in performance between it and the immobilized enzyme was indeed marginal. The results were consistent with the kinetic parameters, indicating that the immobilization method had little effect on the catalytic activity of the enzyme. At the same time, although the free enzyme can reach the reaction limit faster, the immobilized cellulase can convert more of the substrate to glucose. This phenomenon may arise from the progressive decline in the catalytic efficiency of the enzyme throughout the course of the reaction. The protection of the carrier material causes the enzyme activity to decline more slowly, and this additional retained catalytic activity converts more substrates [47].

Thermal stability, pH stability, storage stability, and reusability: Figures S11–S13 show the comparison of the thermal stability, pH stability and storage stability of immobilized and free enzymes. Their half-lives are integrated in Table 3. From the table, it can be observed that both the immobilized enzymes and free enzymes experience some degree of activity loss with the influence of temperature. However, the enzymatic activity loss in immobilized enzymes is significantly lower than that in free enzymes, which becomes more evident under high temperature conditions [47,48].

At the same time, in each experimental group, the immobilized enzymes demonstrate a notably greater pH stability when contrasted with the free enzymes. However, as the pH goes up, the improvement in the stability of the immobilized enzyme consistently diminishes. This may be due to the fact that under alkaline conditions, fewer hydrogen bonds are formed in the solution, and the external liquid is easier to enter the interior of the immobilized enzyme molecule, which reduces the protective performance of the carrier polymer to the enzyme molecule [49].

Figure S12 illustrates the changes in the enzymatic activity for both free and immobilized enzyme solutions that were stored at a low temperature of 4 °C for a duration of 5 weeks. Over the course of one week, the free enzyme loses approximately 18% of its initial enzymatic activity, leading to a final residual activity of 27.6% after being stored for five weeks. In contrast, the immobilized enzyme only experiences a weekly activity loss of about 10%, with a remaining enzymatic activity of 53.6% after five weeks. The results showed that the storage stability of immobilized enzyme was also significantly improved.

The immobilized enzyme was used to catalyze the crushed qualitative filter paper. After a two-hour reaction, the solution was hot-filtered and then cooled to the temperature of 4 °C for 20 min to allow for complete precipitation. The immobilized enzymes were then recovered using low-temperature centrifugation and weighed before being reused in the next cycle. This process was iterated five times, and in each reaction–recovery cycle, the enzyme recovery rate and the enzymatic activity of the recovered immobilized enzyme were assessed. The experiment was conducted in triplicate, and the average value was computed. As illustrated by the results presented in Figure S13, it can be observed that following each cycle, there is an approximately 6% decrease in the enzymatic activity of the immobilized enzyme, while its recovery rate during every catalytic cycle averages to be around 95%. After five cycles, the catalytic activity of the immobilized enzyme was 76.3% of that before the reaction and the weight of the recovered immobilized enzyme was 80.3% of that before the reaction, demonstrating that the immobilized enzyme exhibits a cycling stability for repeated uses.

Experimental catalysis on actual samples: Newspapers, magazines, and corrugated cardboard were crushed into a mixture in a ratio of 1:1:1 as a simulated wastepaper sample for catalytic experiments on actual samples. Under the optimized catalytic conditions mentioned above, with an immobilized enzyme loading 300 mg/L, the solution was monitored every 15 min for its components after a reaction time of one hour at 50 °C and pH = 6, until there was no further glucose concentration increase, ensuring a complete reaction. The efficacy of the reaction was assessed by determining the glucose concentration in the solution following the reaction. After testing, it is found that the glucose concentration in the solution reached 416 mg/L after one hour of reaction, with the glucose concentration peaking at 658 mg/L following a two-hour reaction period, after which it no longer increased. In comparison to controlled laboratory conditions, both the reaction rate and cellulose hydrolysis rate of the catalytic reactions using actual wastepaper samples exhibited a certain degree of reduction. The presence of interfering components such as ink from the printing process hindered the catalytic reaction rate. However, the post-reaction analysis revealed that the glucose concentration in the solution still achieved an 80% efficiency under the laboratory conditions, while 65% of the cellulose in the wastepaper powder was successfully hydrolyzed into glucose, which may be attributed to the relatively low cellulose present in the wastepaper sample. Meanwhile, the solution was hot-filtered, and subsequently allowed to settle at a low temperature of 4 °C for 30 min prior to being centrifuged under similar conditions. As a result, a recovery rate of 90.3% for the immobilized enzymes was achieved.

Taking into account the adsorption capacity of the filter paper as well as the separation efficiency exhibited toward certain immobilized enzyme aggregates and precipitates during the filtration process, it is justifiable to assume that the actual recovery rate of immobilized enzymes would be even higher. This experimental evidence convincingly demonstrates the suitability of this immobilized enzyme for the efficient catalytic hydrolysis of real-world wastepaper.

## 3. Materials and Methods

Materials: The glycinamide hydrochloride, acryloyl chloride, carbon disulfide, and 3-mercaptopropionic acid were purchased from Aladdin Bio-Chem Company. (Shanghai, China). Benzyl bromide, N, N-dimethylacrylamide (DMA), K_2_CO_3_, KOH, HCl, methanol, ethanol, dichloromethane, trichloromethane, anhydrous ether, and 1,4-dioxane were obtained from Macklin Biochemical Co., Ltd. (Shanghai, China). The endoglucanase (EG 3.2.1.4), exoglucanase (CBH 3.3.1.91), and β-D-Glucosidase (EC 3.2.1.21) were synthesized by Hongxun Biotechnology Company (Suzhou, China). All reagents used in this study were of analytical reagent (AR) grade and did not require further purification. All solutions were prepared using deionized water.

Synthesis of PNAGA: The synthesis method of PNAGA is the same as that in reference [42].

Synthesis of P(NAGA-b-DMA): A mixture containing 0.50 g of N, N-dimethylacrylamide (DMA), 1.2 g of PNAGA, 2.00 mg of AIBN, and 2.0 g of 1,4-dioxane was prepared in a 50 mL vessel. Oxygen was eliminated by purging with nitrogen for at least thirty minutes. The blend was then agitated at 70 °C in a nitrogen setting for a duration of 720 min. Following the completion of the reaction, the mixture was cooled to room temperature and briefly frozen at −18 °C. The crude product was precipitated by cautiously adding the viscous reaction liquid obtained after brief freezing into cold diethyl ether. The raw product was dissolved in deionized water and subjected to dialysis with a 10,000 Da dialysis bag to remove residual inorganic salts and a small number of oligomers. Upon completion of dialysis, the product was freeze-dried to yield a white product weighing 1.4 g. ^1^H NMR (D_2_O): δ 1.2–2.4 ppm (3H, -CH_2_-CH-), 3.6–4.4 ppm (2H(N-CH_2_-) + 3H(-N-CH_3_)), 7.6 ppm (5H, ArH).

Preparation of recombinant cellulase LytA enzyme solution: *DH5α E. coli strains* carrying recombinant plasmids pET-CBH-Linker-LytA, pET-EG-Linker-LytA, and pET-Glu-Linker-LytA were purchased and inoculated onto *lb* solid substrate containing kanamycin (working concentration of 1:1000) using the streak plate method. The plates were incubated at a constant temperature for approximately 16 h in a 37 °C incubator, until multiple colonies grew on the surface of the substrate. A full monoclonal colony from the plate was isolated and immersed in 3 mL of *lb* liquid medium containing 50 μg/mL *kanamycin*. The culture was activated by shaking it at 37 °C with 200 rpm for the duration of half a day. Next, 3 mL of the bacterial solution were combined with 300 mL of *lb* medium containing *kanamycin* at a concentration of 50 μg/mL. The mixture was incubated at a constant temperature of 37 °C for a duration of 6 h. Afterward, the bacterial solution was cooled in ice for 20 min, and then, 60 μL of *isopropyl-beta-D-thiogalactopyranoside (IPTG)* inducer was supplemented. The conical flask was transferred to a shaking table and incubated at 25 °C for 16 h. Once the reaction was completed, the solution was centrifuged at a low temperature below 4 °C and a speed of 3000 r/min for 30 min, the supernatant containing the substrate was removed, and the precipitate containing the bacterial cells was retained. The precipitate was washed with 50 mL 50 mM Tris/HCl, pH 8.0 and was resuspended three times; then, the precipitate was collected using centrifugation. The accumulated precipitates were suspended in a solution containing 10 mL of 50 mM Tris/HCl, pH 8.0. The protein inhibitor phenylmethylsulfonyl fluoride (PMSF) was added to a concentration of 1 mM. Subsequently, the mixture was sonicated for 25 min using an ultrasonic disruptor in an ice–water bath. The disrupted solution was centrifuged at 4 °C and 10,000 rpm for 15 min to remove the bacterial precipitates. The supernatant was gathered and carefully transferred into individual 2 mL centrifuge tubes. These tubes were stored at a temperature of 4 °C.

Synthesis of P(NAGA-b-DMA)-cellulase: An aliquot of 100 μL of pre-incubated enzyme mixture, with enzymes in a 1:1:1 volume ratio, was introduced into a centrifugation container, with the P(NAGA-b-DMA) solution added next. The mixture was then oscillated at 60 °C for 120 min. Following the reaction, the mixture was chilled to 4 °C and left undisturbed for 20 min to achieve a thorough precipitation. This was followed by a 20 min low-temperature centrifugation to isolate the supernatant. The resultant precipitate was subjected to freeze-drying to obtain the final product. To explore various conditions for effective immobilization, tests were conducted with different P(NAGA-b-VBA) concentrations (1–5 g/L), pH ranges (4–9), and varying reaction durations (1–3 h). The enzymatic immobilization is calculated using the following equation:

(1) $$\mathrm{Enzymatic}\;\mathrm{immobilization}=\frac{W_{0}-W_{1}}{W}$$

where $W_{0}\;$ (mg) and $W_{1}\;$ (mg) represent the total amount of proteins used for immobilization and unbound proteins, respectively. *W* (mg) represents the mass of the immobilized enzyme P(NAGA-b-DMA)-cellulase.

Means of characterization: ^1^H NMR spectroscopy was utilized to evaluate the makeup of P(NAGA-b-DMA) throughout its synthesis process. GPC was used to measure both the molecular size and its variance. In the GPC setup, the mobile phase used was an aqueous solution containing 0.1 N NaNO_3_, and the parameters were configured for a flow rate of 0.6 mL/min, while the column temperature was maintained at 35 °C. The spectrophotometric measurements allowed for an examination of the upper critical solution temperature behavior exhibited by the polymer as well as the immobilized enzyme. This also enabled the determination of the levels of protein and glucose in the solution. Dynamic light scattering (DLS) and transmission electron microscopy (TEM) were utilized to ascertain the molecular size of the P(NAGA-b-DMA) solution at temperatures both above and below its UCST. FITC-labeled cellulase was used for this purpose. The polymer’s fluorescence, both pre- and post-immobilization, was examined using a laser confocal microscope with an excitation wavelength set at 288 nm. This approach was used to assess the effectiveness of the immobilization process. The scanning electron microscope (SEM) was employed to visualize the surface structures of P(NAGA-b-DMA) and P(NAGA-b-DMA)-cellulase. The compositions of P(NAGA-b-DMA) and P(NAGA-b-DMA)-cellulase were evaluated using a thermogravimetric analysis (TGA) at a rate of 10 °C per minute, ranging from 25 °C to 600 °C, while maintaining a nitrogen atmosphere for protection. Gel electrophoresis was used to investigate the composition of the enzyme in the mixed enzyme solution under incubation [50].

### Protein Concentration and Cellulase Activity Test

The protein concentration was assessed utilizing the BCA assay kit [51]. In this experiment, the test of the enzymatic activity of cellulase was conducted in the following way: Carboxymethyl cellulose (CMC) was dissolved in phosphate buffer solution (PBS) (pH = 5) and configured as a 1% concentration solution. A total of 0.95 mL CMC solution was added to the 10 mL colorimetric tube, 0.05 mL of a certain concentration of cellulase solution to be tested was added to it, and the sample with 0.05 mL PBS added was used as the control group. After shaking evenly, the reaction took place at 50 °C for 10 min. Upon the conclusion of the reaction, 0.2 mL of the reaction mixture was transferred to 0.2 mL of DNS reagent solution for subsequent reaction. This oscillatory reaction continued for 10 min under the condition of 100 °C. After rapid cooling in an ice water bath, and the isochoric volume was made up to 1 mL and allowed to stand for 20 min, and then the absorbance of the solution was measured at 540 nm [52]. The enzyme activity(U) of cellulase was defined as the amount of cellulase required to decompose CMC to produce 1 μmol of glucose in 1 min at 50 °C and pH 5.0.

(2) $$Enzymatic\;activity(U)=\frac{C\times V}{t\times S}$$

Here, C (μmol/L) represents the concentration of the glucose product, V (mL) stands for the aggregate volume of the reaction mixture, t (min) represents the reaction time between enzyme and substrate, and S (mg) represents the total protein content added.

Determination of glucose concentration: The determination methods of the glucose concentration are from the literature [38]. Figure S14 depicts the standard curve of the glucose concentration with a linear regression equation of y = 0.87433x − 0.0643, R^2^ = 0.99867.

Physicochemical properties study of P(NAGA-b-DMA), P(NAGA-b-DMA)-cellulase and free cellulase: The precipitation behaviors of both P(NAGA-b-DMA) and P(NAGA-b-DMA)-cellulase were evaluated by monitoring the transmittance at 470 nm at different temperatures for each solution or suspension. The influence of temperature on the catalytic capabilities of both free and immobilized enzymes was studied at pH 7 across a temperature range of 30–70 °C. Furthermore, the effect of the pH level on the enzymatic activities of both free and immobilized forms was examined at 50 °C within a pH range of 4 to 9. To investigate the temperature stability differences, both free and immobilized enzymes were incubated at temperatures of 30 °C, 50 °C, and 70 °C for a duration of 8 h. Both the free enzyme and the immobilized enzyme were subjected to an 8 h incubation at pH levels ranging from 5 to 7 to assess the disparities in pH stability. The Lineweaver–Burk plot was employed to assess the kinetic characteristics of CMC degradation under optimal reaction conditions, employing both free and immobilized enzymes.

Productivity analysis of immobilized cellulases: A total of 100–300 mg of crushed filter paper was separately added to 10 mL of 1–3 g/L P(NAGA-b-DMA)-cellulase solution and reacted for 6 h at pH = 6 and 50 °C. During the reaction, the glucose concentration in the reaction solution was measured every hour to evaluate the reaction process. After completion of the reaction, the unhydrolyzed filter paper residue was filtered off while still hot, and the filtrate was allowed to settle at a low temperature of 4 °C for 20 min to precipitate the immobilized enzyme. Following its recovery via low-temperature centrifugation, the immobilized enzyme was subjected to freeze-drying and subsequently weighed. To calculate the recovery rate of the immobilized enzyme, the subsequent formula was utilized:

(3) $$Recovery\;of\;immobilized\;enzyme\left(\%\right)=\frac{w}{w_{0}}\times 100\%$$

where *w*_0_ represents the mass of the immobilized enzyme initially added into the reaction system (mg), and *w* represents the mass of the immobilized enzyme recovered and lyophilized after the reaction is completed (mg).

## 4. Conclusions

In this experiment, the UCST temperature-responsive polymer P(NAGA-b-DMA) was synthesized and applied to the immobilization of a cellulase recombinant enzyme with a LytA label as the solid substrate cellulose. The catalytic efficiency of the soluble immobilized enzyme when interacting with a solid substrate significantly outperforms those of traditional insoluble immobilized enzymes acting on insoluble substrates. The calculation of the kinetic parameters indicates that the soluble immobilization method used in this study has little effect on the affinity between the enzyme and substrate, but significantly enhances the reaction rate; the increase in the Michaelis constant is only 11.5%. The immobilized enzyme retained 87.1% of its activity after being incubated at 70 °C for 8 h. Following 5 weeks of storage at 4 °C, 53.6% of the enzyme activity remained. After five cycles of catalysis, the catalytic activity of the immobilized enzyme reached 76.3% of the original dosage, and the amount of recovered immobilized enzyme reached 86.3% of the original dosage, respectively. The immobilization method was effectively employed in the practical degradation experiment involving wastepaper cellulose. The glucose concentration was 658 mg/L after the reaction of mixed wastepaper cellulose was degraded by the immobilized enzyme, and it was 810 mg/L after the reaction, when the substrate was crushed filter paper. The actual degradation rate of the wastepaper reached 80% in the laboratory environment, and the recovery rate of immobilized enzyme was 90.3%.

## Figures and Tables

**Table 1:** Molecular weight and polydispersity of synthesized P(NAGA-b-DMA).

No.	Molecular Formula	Mw (Feed) g/mol	Mw (Polymer) g/mol	Polydispersity (PDI)
UCST-1	P(NAGA_100_-b-DMA_5_)	13,567	16,774	1.485312
UCST-2	P(NAGA_150_-b-DMA_5_)	19,967	23,514	1.368441
UCST-3	P(NAGA_200_-b-DMA_5_)	26,367	34,642	1.374864
UCST-4	P(NAGA_100_-b-DMA_10_)	14,062	17,685	1.354778
UCST-5	P(NAGA_100_-b-DMA_15_)	14,557	17,254	1.327863
UCST-6	P(NAGA_100_-b-DMA_20_)	15,052	19,256	1.474235

**Table 2:** Kinetic parameters of free cellulase and immobilized cellulase.

Carrier Material	$K_{m}$ (mM)		$K_{cat}$ (s^−1^)		$K_{cat}/K_{m}$ (s^−1^·mM^−1^)	
	Free Enzyme	Immobilized Enzyme	Free Enzyme	Immobilized Enzyme	Free Enzyme	Immobilized Enzyme
P(NAGA-b-DMA)	0.531	0.592	7230.667	5008.805	13,617.07	8460.819
chitosan/magnetic porous biochar [45]	8.298	12.134				
Fe_3_O_4_@C magnetic nanoparticles [46]	0.59	2.42	0.124	0.058	0.21	0.024

**Table 3:** Thermal stability, pH stability, and storage stability of free enzyme and immobilized enzyme.

	Half-Life Period of Free Enzyme	Half-Life Period of Immobilized Enzyme
Temperature stability (h)		
30 °C	35.3	106.5
50 °C	20.1	41.9
70 °C	12.5	31.3
pH stability (h)		
pH = 5	46.5	97.1
pH = 6	60	90.4
pH = 7	25.4	30.9
Storage stability (week)		
4 °C	3.2	5.4

## Data Availability

Data are contained within the article and supplementary materials.

## Supplementary Materials

The following supporting information can be downloaded at: https://www.mdpi.com/article/10.3390/molecules29051039/s1, Figure S1. ^1^H NMR data of each step of the product in the experiment; Figure S2. DLS data of P(NAGA-b-DMA); Figure S3. Effect of different conditions on the UCST of P (NAGA-b-DMA) (a) Polymer concentration (b) Degree of polymerization of NAGA (c) Degree of polymerization of DMA (d) Urea concentration; Figure S4. SDS-PAGE analysis of mixed enzyme solution, Swimlane M: Marker, Swimlane 1: supernatant containing empty carriers, Swimlane 2: supernatant containing mixed enzyme solutions; Figure S5. Optimal immobilization reaction pH (a) and temperature (b); Figure S6. Effect of Immobilization Reaction Time on Enzyme Immobilization Capacity; Figure S7. TGA data of P(NAGA-b-DMA) and P(NAGA-b-DMA)-cellulase; Figure S8. Effect of different conditions on the UCST of P (NAGA-b-DMA)-cellulase (a) Polymer concentration (b) Degree of polymerization of NAGA (c) Degree of polymerization of DMA (d) Urea concentration; Figure S9. (a) The Relative activity of free cellulase and P(NAGA-b-DMA)-cellulase placed at 30–70 °C, (pH = 6). (b) The Relative activity of free cellulase and P(NAGA-b-DMA)-cellulase placed at 50 °C, (pH = 4–8); Figure S10. The Lineweaver Burk plots of free cellulase and P(NAGA-b-DMA)-cellulase; Figure S11. Temperature stability and pH stability of free cellulase and P(NAGA-b-DMA)-cellulase (a) 30 °C (b) 50 °C (c) 70 °C (d) pH = 5 (e) pH = 6 (f) pH = 7; Figure S12. Storage stability of P(NAGA-b-DMA)-cellulase; Figure S13. Recycling stability of P(NAGA-b-DMA)-cellulase; Figure S14. Standard curve of glucose concentration; Table S1. The influence of different polymer composition and polymer solution concentration on loading capacity and recovery rate.
