# Supplementary material for: UCST-Type Soluble Immobilized Cellulase: A New Strategy for the Efficient Degradation and Improved Recycling Performance of Wastepaper Cellulose
Source: Molecules. 2024 Feb 28;29(5):1039. doi: 10.3390/molecules29051039 (PMC10934508; doi:10.3390/molecules29051039)
Supplement: Supplementary file 1 [file molecules-29-01039-s001.zip › molecules-2882566-supplementary.pdf]

Optimization of immobilization conditions for P(NAGA-b-DMA)-cellulase. Due to the enzyme solution being a mixture obtained from fermentation and cultivation, it is not possible to obtain precise enzyme dosage. The ratio of polymer to immobilized enzyme in immobilized condition is achieved by adding 1-4 g/L polymer to 2 mL mixture of enzyme solution. Based on previous research findings, it has been observed that the molecular weight of immobilized enzyme increased rapidly due to the introduction of enzyme, resulting in an elevated UCST. In order to enhance the immobilization capacity of enzyme while maintaining its UCST performance, the pre-immobilized polymer's UCST needs to be as low as possible. Therefore, UCST1/4/5/6 with NAGA block polymerization degree of 100 was selected for immobilization reaction, and the reaction was carried out at pH=5 and 60 ℃ for 2 hours.

As shown in table S1, with the rise in concentration of the added polymer solution, the enzymatic immobilization capacity gradually decreases. Based on previous experimental findings, it can be interfered that higher concentrations of polymer solutions have a higher UCST. After immobilization reaction with the enzyme, high-concentration polymers continue to raise their cloud point above the temperature of immobilization reaction. At this point, the aggregation and precipitation of immobilized enzyme significantly reduces the reaction, resulting in a decrease in enzymatic immobilization capacity. In the case of constant NAGA block copolymerization degree, as the DMA block copolymerization degree increases, there is a significant increase in enzymatic immobilization capacity. However, correspondingly, there is also a noticeable decrease in enzymatic activity. This may be due to a large amount of DMA blocks covering the active site of the enzyme while immobilizing the enzyme. Under the premise of comprehensive consideration of the enzymatic immobilization capacity and enzymatic activity retention, 2g/L UCST-5 was chosen for the subsequent immobilization reaction.

As depicted in Fig.S5a, the immobilization reaction was carried out at 60 ℃ and pH=3-7 for two hours. It can be observed that with the increasing alkalinity of the solution, there is a certain degree of enhancement in enzymatic immobilization

capacity. This is because under relatively alkaline conditions, hydrogen bonds in the solution are weaker, allowing the polymer to dissociate better in water. The enzymatic activity of immobilized enzyme is the highest at pH=5, as more acidic conditions may not be suitable for the survival of cellulase, and result in a significant decrease in enzymatic activity. However, at pH=6, the decrease in enzymatic activity compared to pH=5 is minimal, while the enzymatic immobilization capacity has been improved to a certain extent. Therefore, the pH for this immobilization reaction is set at 6.

Fig.S5b illustrates the effect of reaction temperature on the immobilization reaction. The immobilization capacity of enzyme increases to a certain extent with an increase in temperature. This is because, after the polymer undergoes immobilization reaction with a sufficient amount of enzyme, the UCST of immobilized enzyme rises above the reaction temperature. At this point, the immobilized enzyme agglomerates and precipitates, significantly reducing its participation rate in the immobilization reaction. However, at higher temperatures, they can continue to participate in the reaction and achieve higher enzymatic immobilization. Nevertheless, considering that there is not much increase of immobilization rate beyond 60 °C, and that high temperatures lead to significant loss of enzymatic activity, it is still optimal to set the immobilization temperature at 60 °C.

The immobilization reaction was conducted under the condition of 60 °C and pH=6. After 30 minutes, the immobilization capacity of enzyme was measured every 10 minutes. As depicted in Fig.S6, by the time of 70 minutes, the reaction was essentially completed.

Optimal catalytic conditions of free cellulase and P(NAGA-b-DMA) cellulase. To optimize the optimal catalytic conditions of immobilized enzyme, a catalytic reaction was conducted using both free and immobilized cellulase in a buffer solution at pH=6, and the substrate used was CMC. The reaction temperature was set between 30 to 70 °C respectively. The data obtained on relative enzymatic activity of free enzymes and immobilized enzymes are depicted in Fig.S9a. The optimal catalytic temperature for free cellulase is 50 °C, and its catalytic activity decreases when the temperature is below or above 50 °C. The optimal catalytic temperature for immobilized enzyme is

also 50 °C, but the range of catalytic temperatures widens, and the enzymatic activity decreases only slightly at 60 °C. The activity of immobilized enzyme was consistent with that of free enzyme. It was proved that immobilization had no significant effect on enzyme performance. At temperatures below 50 °C, low temperatures are unable to activate the enzymatic activity fully, resulting in lower enzymatic activity. When the temperature exceeds 50 °C, the excessively high temperature leads to enzymatic inactivation, resulting in a decrease in measured activity. Due to the covering of the carrier polymer on the enzyme, the active site of the enzyme is protected, the carrier polymer acts as a layer of insulation, which makes the microenvironment near the active site of the enzyme change slowly, making the enzyme insensitive to changes in the external environment, so it retains more catalytic activity at high temperatures.

Under the condition of pH=4-8, free and immobilized cellulase were used to catalyze the substrate CMC at 50 °C for a certain period of time, and the relative enzymatic activity at each pH value was calculated according to the measured maximum enzymatic activity of 100%. As depicted in Fig.S9b, the free enzyme exhibited the maximum catalytic activity at pH=5, while the immobilized enzyme demonstrated the highest catalytic activity at pH=6. Meanwhile, under the condition of constant pH fluctuation range, the relative enzymatic activity of immobilized enzyme decreases at a slower rate compared to free enzymes. This is because the polymer encapsulates the enzyme, resulting in a slower change in the microenvironment surrounding the enzyme and making it less sensitive to pH variations. Therefore, for subsequent catalytic reactions, the pH value was adjusted to 6.

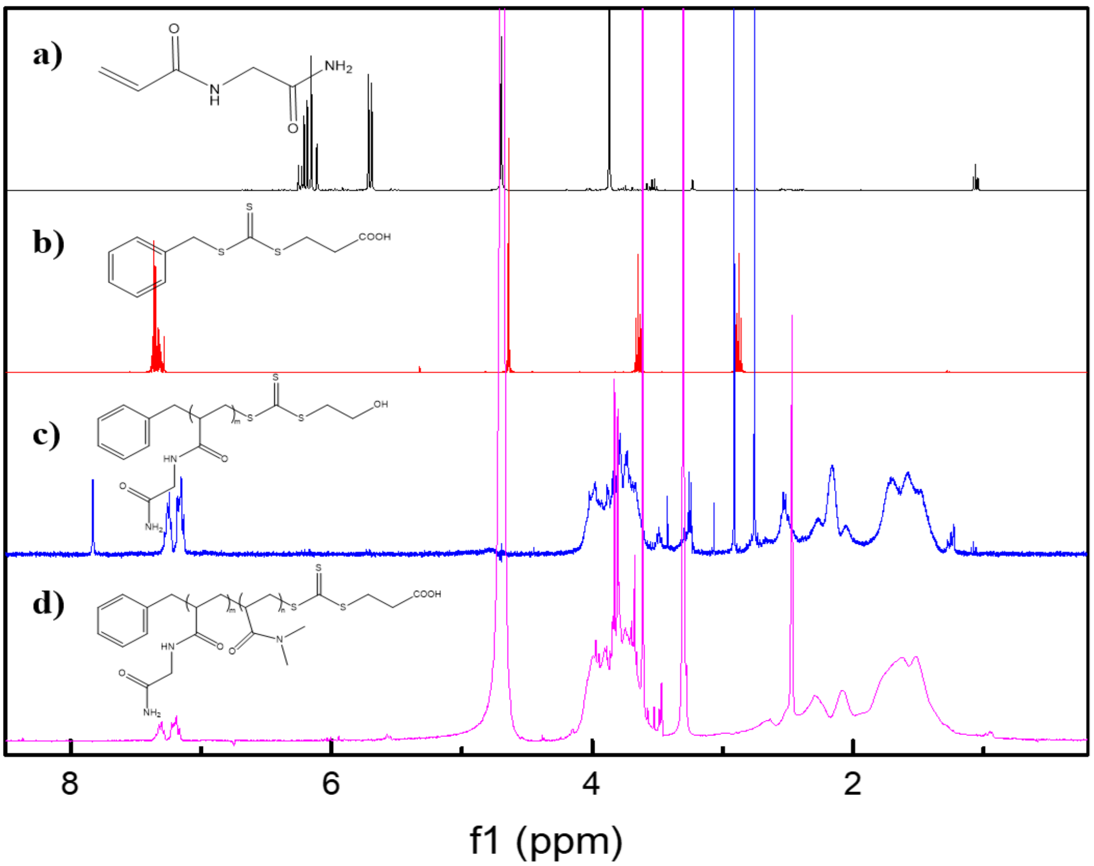

Figure.S1 ^{1}H NMR data of each step of the product in the experiment

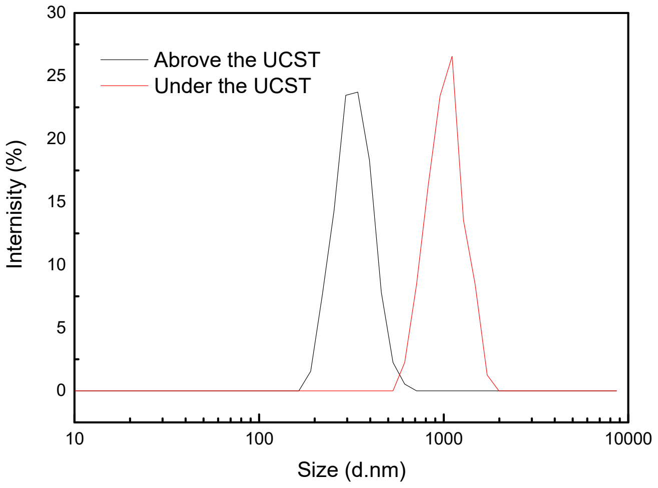

Figure.S2 DLS data of P(NAGA-b-DMA)

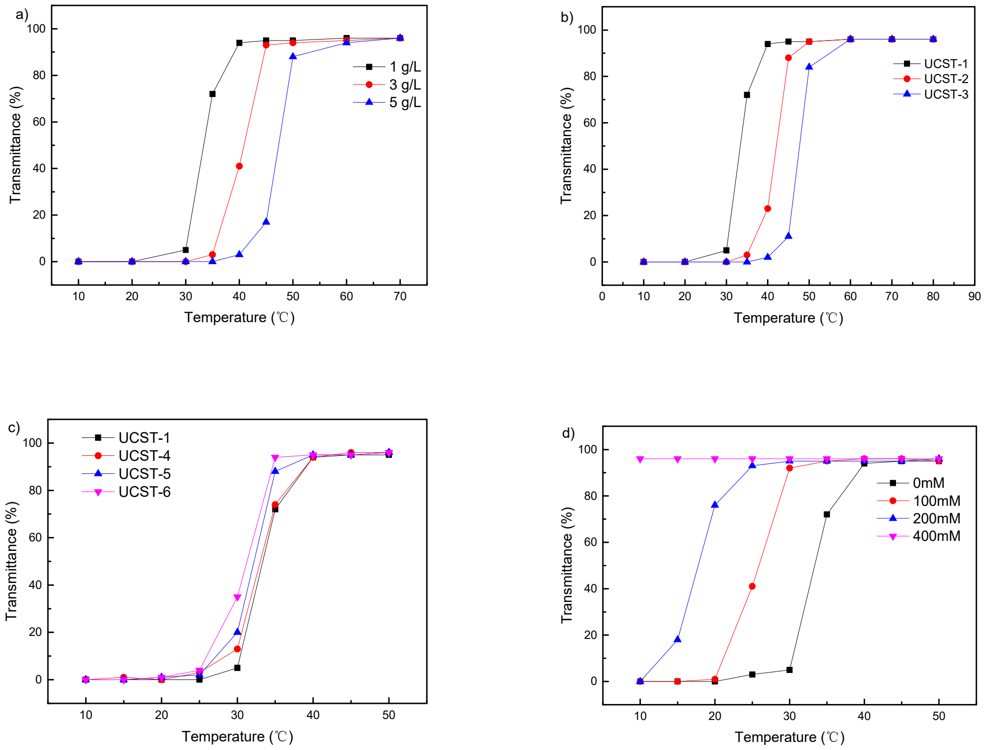

Figure.S3 Effect of different conditions on the UCST of P (NAGA-b-DMA) (a) Polymer concentration (b) Degree of polymerization of NAGA (c) Degree of polymerization of DMA (d) Urea concentration

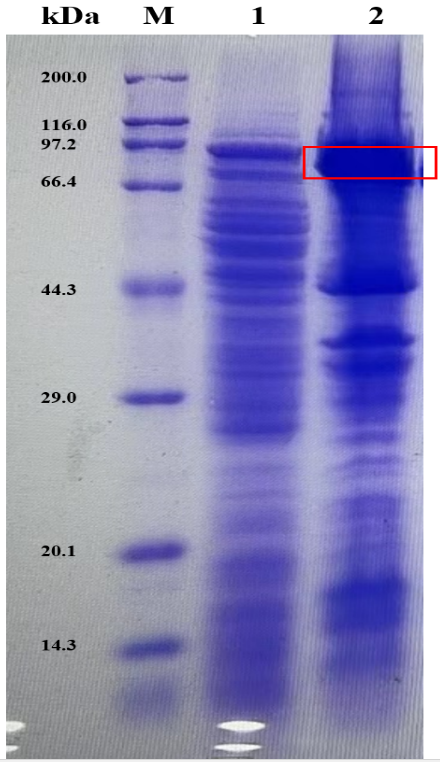

Figure.S4 SDS-PAGE analysis of mixed enzyme solution, Swimlane M: Marker, Swimlane 1:

supernatant containing empty carriers, Swimlane 2: supernatant containing mixed enzyme solutions.

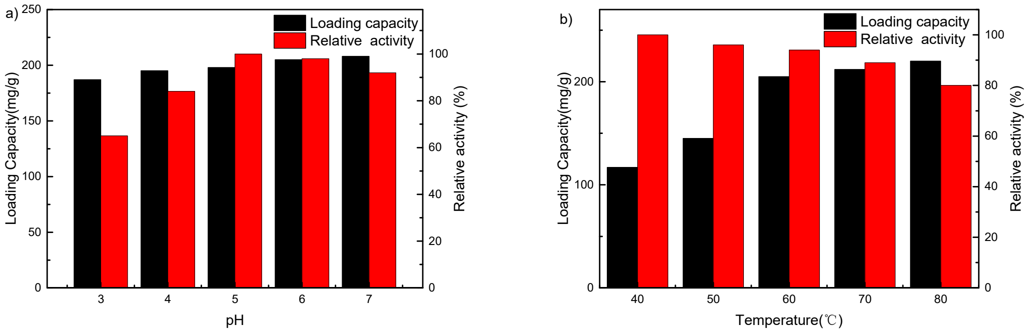

Figure.S5 Optimal immobilization reaction pH (a) and temperature (b)

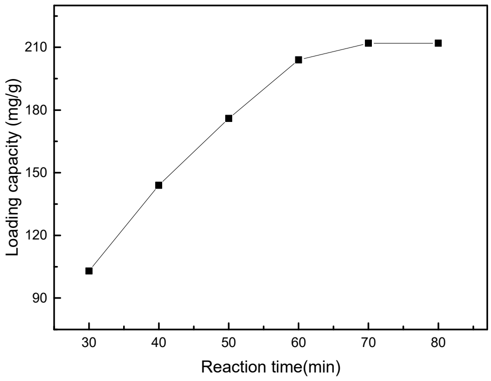

Figure.S6 Effect of Immobilization Reaction Time on Enzyme Immobilization Capacity

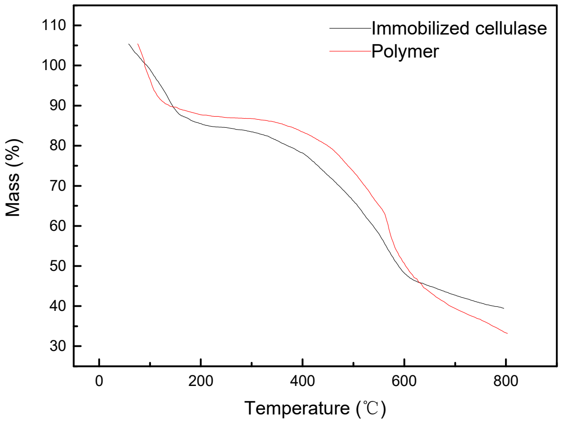

Figure.S7 TGA data of P(NAGA-b-DMA) and P(NAGA-b-DMA)-cellulase

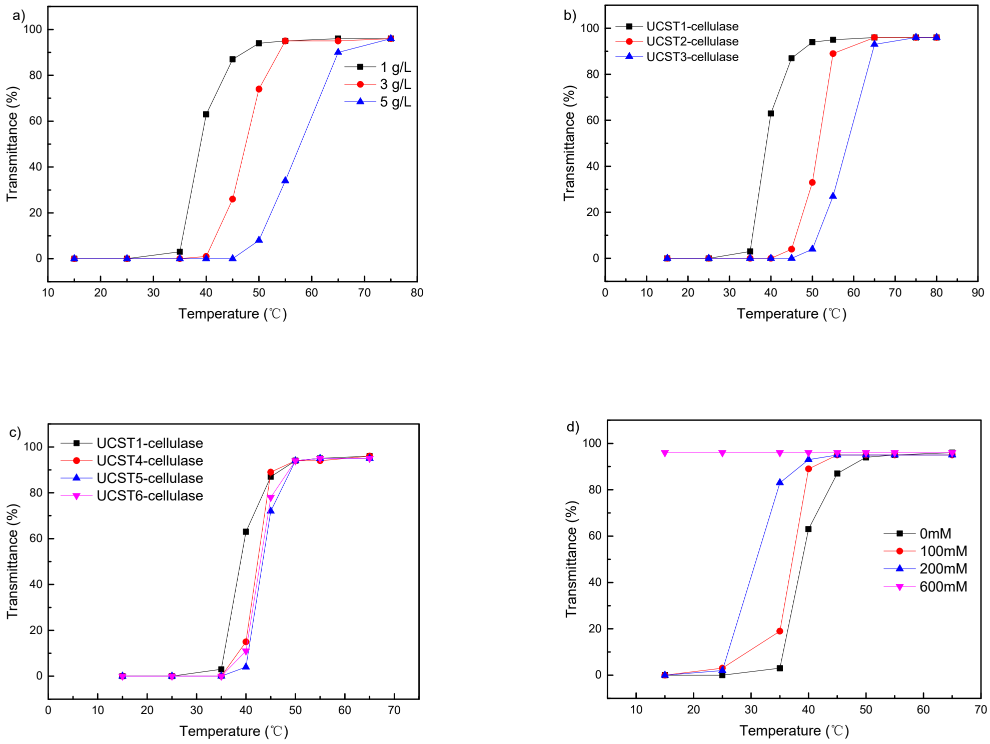

Figure.S8 Effect of different conditions on the UCST of P (NAGA-b-DMA)-cellulase (a) Polymer concentration (b) Degree of polymerization of NAGA (c) Degree of polymerization of DMA (d) Urea concentration

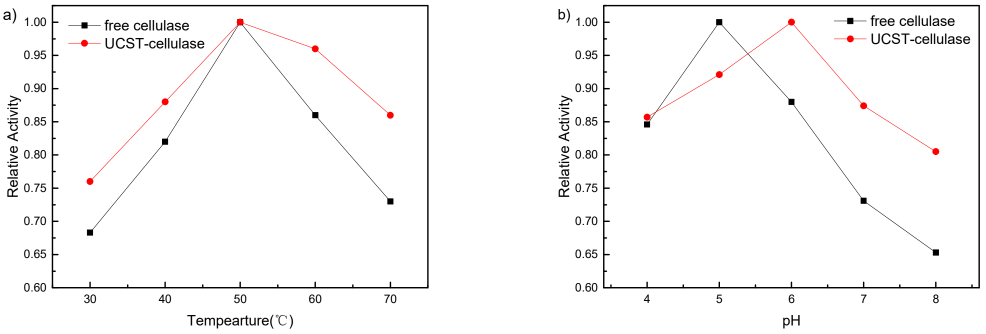

Figure.S9 (a)The Relative activity of free cellulase and P(NAGA-b-DMA)-cellulase placed at 30-70℃, (pH=6). (b)The Relative activity of free cellulase and P(NAGA-b-DMA)-cellulase placed at 50℃, (pH=4-8)

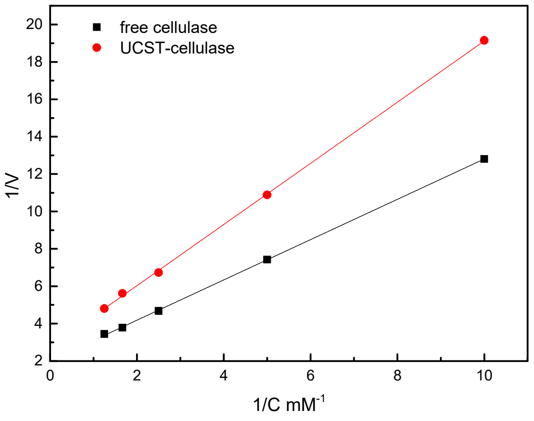

Figure.S10 The Lineweaver Burk plots of free cellulase and P(NAGA-b-DMA)-cellulase.

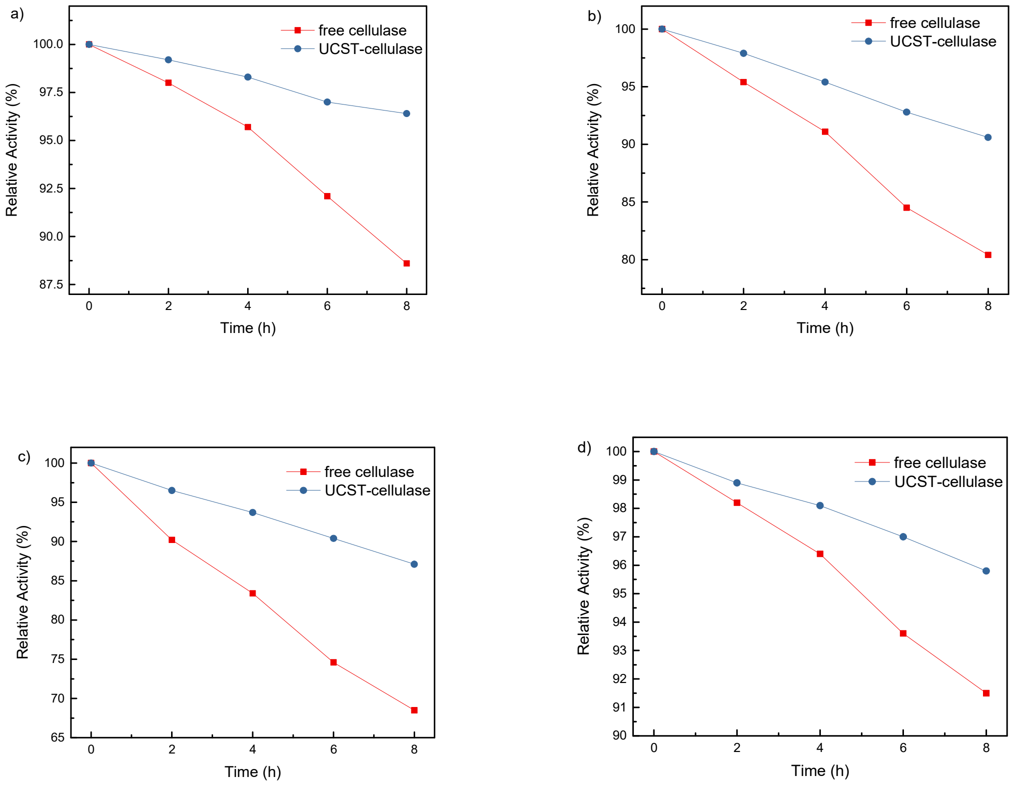

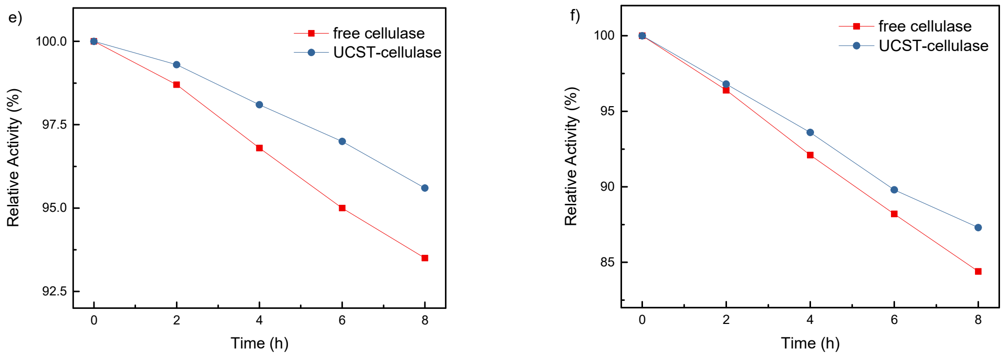

Figure.S11 Temperature stability and pH stability of free cellulase and P(NAGA-b-DMA)-cellulase (a)30℃ (b)50℃ (c)70℃ (d)pH=5 (e)pH=6 (f)pH=7

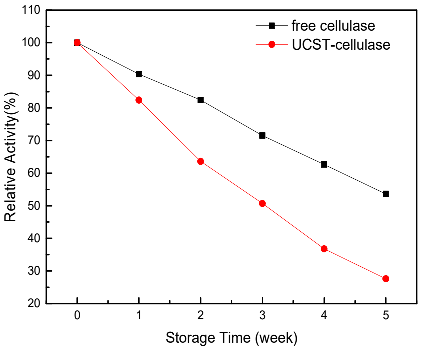

Figure.S12 Storage stability of P(NAGA-b-DMA)-cellulase

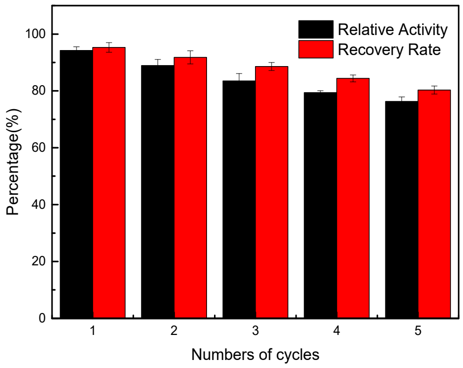

Figure.S13 Recycling stability of P(NAGA-b-DMA)-cellulase

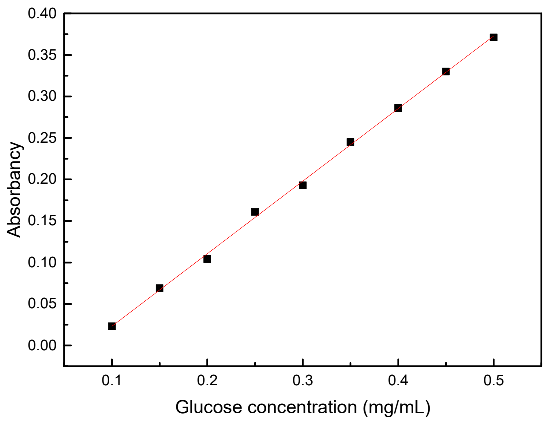

Figure.S14 Standard curve of glucose concentration

Table. S1 The influence of different polymer composition and polymer solution concentration on loading capacity and recovery rate

Sample number	Polymer number	Polymer concentration(g/L)	Loading capacity (mg/g)	Relative enzyme activity(%)
1	UCST-1	1	85	97
2	UCST-4	1	135	95
3	UCST-5	1	205	88
4	UCST-6	1	232	84
5	UCST-1	2	79	98
6	UCST-4	2	134	94
7	UCST-5	2	198	90
8	UCST-6	2	204	87
9	UCST-1	3	68	98
10	UCST-4	3	120	95
11	UCST-5	3	155	93
12	UCST-6	3	191	90
13	UCST-1	4	54	100
14	UCST-4	4	82	98

15	UCST-5	4	125	96
16	UCST-6	4	137	92
